# Tailoring a Silver Paste for Additive Manufacturing of Co-Fired Ferrite Magnetic Components

**DOI:** 10.3390/ma12050817

**Published:** 2019-03-11

**Authors:** Lanbing Liu, Chao Ding, Yunhui Mei, Guoquan Lu

**Affiliations:** 1 The Department of Materials Science and Engineering, Virginia Tech, Blacksburg, VA 24061, USA; lanbing@vt.edu (L.L.); dingchao@vt.edu (C.D.); 2 School of Materials Science and Engineering, Tianjin University, Tianjin 300072, China; yunhui@tju.edu.cn; 3 The Bradley Department of Electrical and Computer Engineering, Virginia Tech, Blacksburg, VA 24061, USA

**Keywords:** additive manufacturing, co-fire, shrinkage mismatch, ferrite magnetic components

## Abstract

Additive manufacturing (AM), or 3D-printing, has the potential for rapid prototyping of innovative designs of magnetic components used in power electronics converters. In this study, we tailored a silver paste as the metal feedstock of an extrusion 3D printer so that the metal would be compatible with a ferrite paste feedstock for 3D-printing of ferrite magnetic components. We focused on adjusting the metal formulation to match its shrinkage to that of the ferrite and to improve adhesion during the co-sintering process of the printed part. We found that a 5 wt % addition of ferrite powder in the metal paste can achieve matched shrinkage and strong adhesion. Evaluation of the co-sintered magnetic components showed no significant defects, such as cracks, warpage, or delamination, between the metal and ferrite. The shear strength between the two sintered materials was greater than 50 MPa, and the electrical resistivity of the sintered metal winding was less than twice that of the bulk silver, which is lower than those of most 3D-printed winding metals reported in the literature.

## 1. Introduction

Magnetic components are necessary elements in power converters and are often bulky and lossy. One way to reduce the magnetic size is to employ novel designs [1,2,3,4,5,6,7,8] of the magnetic core to improve inductance density or reduce loss. Today, cores are fabricated by pressure-assisted molding followed by heat treatment, and metal wires are then hand-wound or machine-wound around the cores. These manufacturing methods lead to a limited number of standard inductor and transformer structures available on the commercial market. However, fabricating innovative magnetic components aimed at shrinking the size or reducing loss of the structures would be too costly and time-consuming.

Due to its versatility in shaping complex geometries, additive manufacturing (AM), or 3D-printing, has the potential to fabricate novel magnetic core structures. Furthermore, it can produce newly designed prototypes in days or hours for quick verification. There are a small number of studies [9,10,11,12,13,14,15,16,17,18] reported in the literature regarding 3D-printed magnetic materials, and even fewer on 3D-printed magnetic components (such as magnetic cores and metal windings). All of the studies examined, except one, reported that 3D-printed magnetic materials had a low relative permeability of less than 10, which is too low for power electronics (PE) applications. Yan et al. [17] fabricated several designs of power inductors by 3D-printing both the magnetic core and metal windings. They formulated an iron powder paste and combined it with a commercial silver paste to construct inductors in a multi-extrusion 3D printer. After printing, they used a low-temperature (<250 °C) curing process to solidify the magnetic paste and sinter the silver paste. The relative permeability of their magnetic core was 25, and the winding resistivity was about 3 times that of bulk silver. The lack of more studies like Yan et al.’s can be attributed to a lack of compatible high-performing magnetic and metallic feedstock materials. This is especially so if one is to make ferrite components for which the core and winding materials have to be sintered together at elevated temperatures.

Ferrite inductors are the first choice for making high-frequency power converters because ferrite cores generally have low core losses and high permeabilities in and beyond megahertz range [19,20]. There are two types of ferrite materials widely used in the power electronics industry for making inductors: MnZn ferrite and NiZn ferrite. MnZn ferrites usually have relative permeability values ranging from 1000 to 10,000, and lower core-loss density than NiZn ferrite at <5 MHz. The relative permeability of NiZn (including NiCuZn) ferrites are usually <500 but they have lower core-loss density for frequencies >5 MHz. Selecting one type over the other for a power converter also requires the designer to consider other factors such as maximum flux density, operating temperature, thermal conductivity, availability of size, and geometry. To the best of our knowledge, no one has reported 3D-printing high-performance ferrite components for PE applications. This stems from the complexities of processing the components, specifically the sintering or firing of the ceramic and metal materials in the state of a porous powder compact at over 800 °C. In general, ceramic and metal powder compacts have different shrinkage profiles of densification, leading to cracks, camber or warpage, or delamination in the finished parts [21,22,23,24,25,26].

In this study, we focused on engineering the formulation of a silver paste feedstock so that its shrinkage profile, upon sintering, would match that of a ferrite feedstock. We reported the development of the NiCuZn ferrite feedstock elsewhere [27,28] and showed that the relative permeability of the ferrite core sintered at <1000 °C can be tailored between 10 and 100, and the core-loss densities were similar to those of commercial NiZn ferrite cores having similar permeabilities. The objective of this work was to explore the potential of co-printing and co-sintering the ferrite feedstock with a silver feedstock to make ferrite inductors. To mitigate the shrinkage mismatch between the two types of materials, small fractions of the ferrite powder were blended into a commercial silver paste. The effects of ferrite addition in the silver paste on metal shrinkage were measured, on the quality of the co-sintered parts was evaluated, and on the adhesion between the two co-sintered materials was characterized. In addition, the electrical and magnetic properties of the sintered silver feedstock were determined.

## 2. Materials and Methods

### 2.1. The 3D-Printing Platform

**Figure 1:**
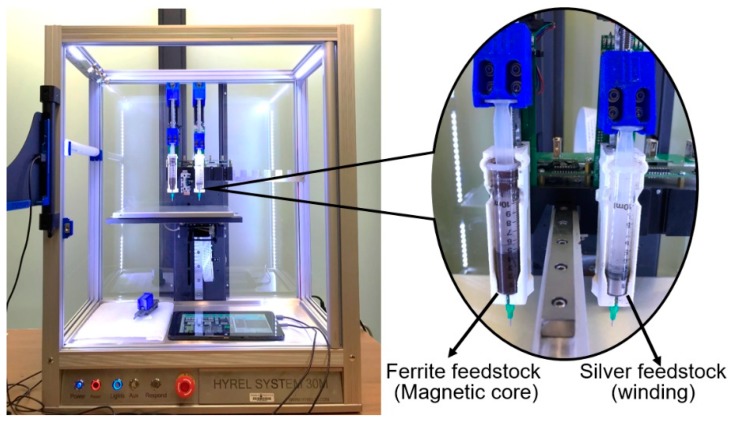
The multi-extrusion 3D printer used in this study with two of its four extruders installed, one with a ferrite feedstock and the other with a silver feedstock.

The 3D-printing platform used in this study was a multi-extrusion 3D printer (System 30M, Hyrel International, Norcross, GA, USA). It is particularly suitable for processing multiple materials from paste form into a single object [17]. Figure 1 shows the 3D printer with two of its four extruders installed, one with a ferrite feedstock and the other with a silver feedstock.

### 2.2. Preparation of Ferrite and Silver Feedstock

The ferrite feedstock was prepared by mixing a commercial NiCuZn ferrite powder (LSF90, Powder Processing & Technology, LLC, Valparaiso, IN, USA, average particle size 0.8 μm), monomer binder (pentaerythritol tetraacrylate, Sigma-Aldrich Co. LLC, St. Louis, MO, USA), and diluent (polyethylene glycol 400, Alfa Aesar, Haverhill, MA, USA). The weight ratio of ferrite powder to monomer binder was 19. The solid loading (fraction of ferrite powder in the feedstock) was 78 wt %.

The silver feedstock was made by tailoring a commercial silver paste (nanoTach-X, NBE Tech, LLC, Blacksburg, VA, USA). The addition of ceramic powders into metal paste has been shown to be effective in mitigating shrinkage mismatch between ceramic and metal layers during co-firing of multilayer monolithic thick films [22,23,24]. Thus, to adjust the shrinkage of the silver feedstock, LSF90 ferrite powders were added at different concentrations of 1 wt %, 5 wt %, and 10 wt % into the silver paste. To homogenize the mixture in a container, zirconia milling media of 5 mm diameter and at around 1:1 media-to-content weight ratio was added and then shaken for 5 min in a high-speed vibrating ball miller (MSK-SFM-3, MTI Corp., Richmond, CA, USA).

### 2.3. Measurements of Densification Shrinkage

**Figure 2:**
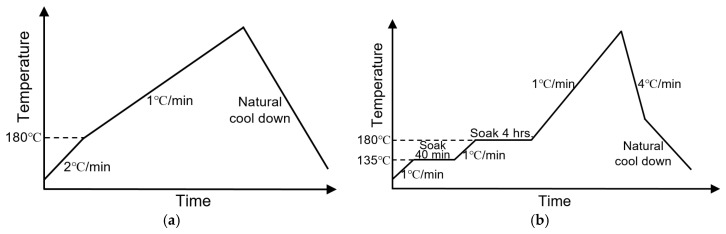
Heating profiles used for measuring the shrinkage of (**a**) silver feedstock and (**b**) ferrite feedstock.

Shrinkage of the two types of feedstock was measured by monitoring their dimensional changes as they underwent densification. Ferrite samples, 10 mm × 10 mm × 1 mm in size, were made by printing the ferrite feedstock in the 3D printer while the silver samples of 8.5 mm diameter and 0.5 mm thickness were made by stencil-printing. For each feedstock, the plot of shrinkage versus temperature was obtained by measuring the shrinkage of several samples, using one sample for each temperature point. Each sample was heated to a specified temperature, and then cooled to room temperature. The dimensions of the sample before and after the heat treatment were measured by ImageJ [29] and the shrinkage at that temperature was calculated. The samples were sintered in a furnace following the heating profiles shown in Figure 2: (a) for the silver and (b) for the ferrite. The maximum sintering temperatures of the heating profiles varied between 300 and 930 °C, thus, they are not indicated in figures. As shown in Figure 2b, the ferrite feedstock was soaked at 135 °C to polymerize the monomer and solidify the feedstock, and then soaked at 180 °C to remove most of the organic components. To prevent cracking, the low heating rate of 1 °C/min was used to slow down outgassing of the ferrite. The heating profile of the silver feedstock, as shown in Figure 2a, was slightly adjusted for heating to 180 °C. This adjustment was mainly to save processing time because we found no difference in the metal shrinkage if we used the same heat-and-hold steps to 180 °C as used for the ferrite. The removal of organic components in the silver paste was much quicker because the organics are more volatile and silver metal winding samples were much thinner than the ferrite core samples.

The profile in Figure 2b, was also the co-sintering profile of silver and ferrite feedstock, with a peak sintering temperature of 930 °C (soak for 2 h). The peak sintering temperature was set as 930 °C because of the melting point of silver (962 °C).

### 2.4. Measurements of the DC Electrical Resistivity of Sintered Silver

The DC electrical resistivity of sintered silver from the metal feedstock was measured using the four-point probe method. The test samples, 40 mm × 1 mm × 90 μm thick strips, were made by stencil-printing each formulation of the silver paste, followed by sintering at 930 °C with a 2 h soak and a ramp-up profile similar to that shown Figure 2a. For each feedstock, three samples were characterized, and the average and standard deviation are listed in Table 1.

### 2.5. Measurements of Relative Permeability

We also measured the relative permeabilities of the sintered silver from the metal feedstock. Since the silver feedstock contained some amounts of the ferrite powder, we were interested in the effect of the magnetic particles on the magnetic property of the winding material. The test samples for the magnetic measurements were 3D-printed toroid cores with an outer diameter of about 11 mm, inner diameter of roughly 6 mm, and thickness of approximately 0.6 mm. Printing was followed by sintering at 930 °C with a 2 h soak and ramp-up profiles in Figure 2. The toroid cores were wire-wound into inductors, and their inductances were measured over a frequency range from 100 Hz to 2 MHz at zero DC bias using a precision impedance analyzer (4294A; Agilent, Santa Clara, CA, USA) with a test fixture (16047E; Agilent, Santa Clara, CA, USA). Then, the relative permeabilities were determined from the measured inductances and finite element analysis (FEA) simulations of the inductor structures with core relative permeabilities that would result in the measured inductances. 

Each plot of measured inductance versus frequency showed a plateau at low frequencies (roughly <20 kHz), and the inductance then dropped with increasing frequency. This was because large eddy currents were generated in the silver toroid samples when they were measured at high frequencies. Thus, the plateau inductance values were used to determine the permeabilities. For each feedstock formulation, the average relative permeability and standard deviation were obtained by analyzing three samples, and are listed in Table 2.

### 2.6. Measurements of Ferrite–Silver Bonding Strength

The adhesion strength between the two materials of the ferrite component was characterized by measuring the shear strength between them. Test samples were made by 3D-printing the ferrite feedstock into cuboids of dimension 5 mm × 8 mm × 1.5 mm and then printing parallel lines of the as-received silver paste and the silver feedstock with 5 wt % ferrite powder on top of the ferrite. The length, width, and thickness of the silver lines were around 5, 0.7, and 0.8 mm, respectively. After the printed samples were co-sintered at 930 °C with a 2 h soak and the ramp-up profile shown in Figure 2b, the adhesion strength between the two materials was measured by shearing the silver lines in a Dage 4000 bond tester under displacement control at a loading speed of 0.1 mm/s with a shear height of 50 μm. The shear strength was calculated by taking the ratio of the maximum load-before-failure over the shear area.

## 3. Results and Discussion

**Figure 3:**
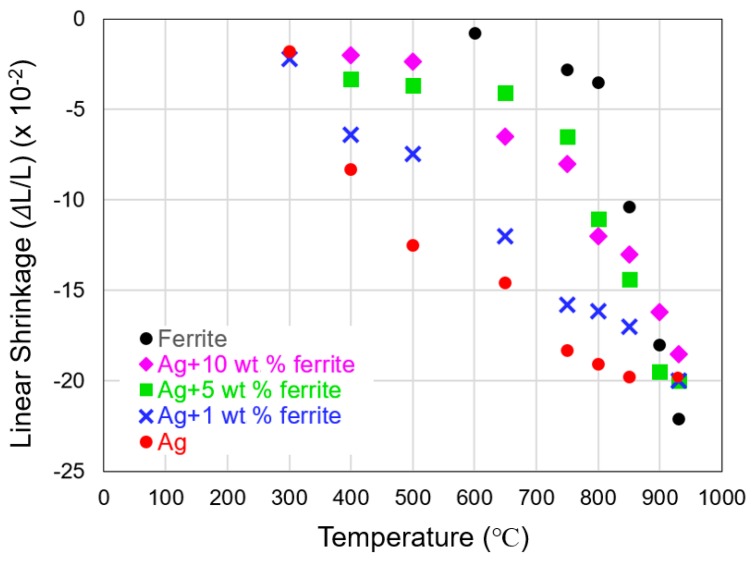
Plots of linear shrinkage of the ferrite feedstock and four silver pastes after sintering at temperatures between 300 and 930 °C.

Figure 3 shows the linear densification-shrinkage profile of the ferrite feedstock and those of four silver pastes after sintering at peak temperatures between 300 and 930 °C. The ferrite and the as-received silver paste had significantly mismatched profiles. The silver paste started to shrink at <300 °C, and had shrunk nearly 15% at around 600 °C before the ferrite feedstock started to shrink. Over most of the temperature range, the silver shrank far more than the ferrite. However, by adding ferrite powder into the silver paste, the shrinkage mismatch was drastically reduced. Of the three silver pastes with 1 wt %, 5 wt %, and 10 wt % ferrite powder, the sample with 5 wt % ferrite powder had a shrinkage profile that most closely matched to that of ferrite.

**Figure 4:**
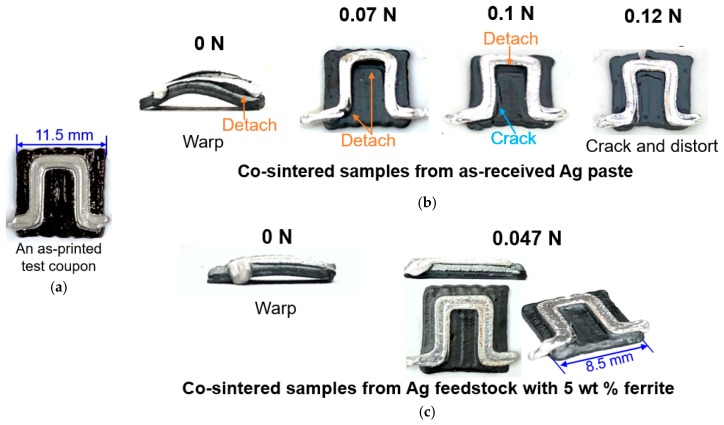
(**a**) A 3D-printed test coupon with the ferrite feedstock and modified silver feedstock having 5 wt % ferrite. Co-sintered test coupons from (**b**) as-received Ag paste and (**c**) Ag feedstock with 5 wt % ferrite, with different weights of alumina plates placed on top throughout the co-sintering process.

To show the effect of better-matched densification-shrinkage profiles on the quality of co-sintered ferrite and silver parts, test coupons like the one shown in Figure 4a were 3D-printed. We first printed the ferrite feedstock to form a square plate with side length of around 11.5 mm and thickness of around 0.8 mm. Then, the silver feedstock with 5 wt % ferrite powder, or the as-received silver paste, was printed in a quasi-Π shaped pattern on top of the ferrite plate. The test coupon structure is similar to half of a one-turn planar inductor [18,30,31,32,33,34]. After the coupons were printed, they were co-sintered in air at 930 °C with a 2 h soak following the profile showed in Figure 2b. Initially, all of the coupons after co-sintering, as shown in the left-most column in Figure 4b labeled by 0 N, had distortion or warpage. Those made with the as-received silver paste also showed detachment of silver from the ferrite plate. The coupons made with the modified silver feedstock showed no delamination and had significantly less warpage due to the better-matched shrinkage profiles. To reduce warpage, alumina plates of different weights were placed on top of the coupons as they underwent co-sintering. This is a common practice in the firing of ceramic parts. For test coupons made with the as-received silver paste, applying the weight reduced warpage but did not eliminate detachment between the silver and ferrite, as shown in the top row of Figure 4b, labeled as 0.07 N and 0.1 N. Further increasing the weight can eliminate detachment but generate cracks and distortion on the ferrite, as shown in the top row of Figure 4b labeled as 0.12 N. However, for the coupons made with the modified silver feedstock, only 0.047 N in weight was enough to eliminate warpage without generating cracks or distortion on the ferrite, as shown in the bottom row of Figure 4b. The co-sintering tests showed that the modified silver paste has the potential for making high-quality ferrite inductors by the co-extrusion and co-sintering process.

In Section 2.6, we described the structures and dimensions of samples for testing shear strength between the co-sintered silver and ferrite. No shear data were collected from the samples made with the as-received silver paste since the silver lines detached from the ferrite cuboid after co-sintering. Regarding the samples made with silver paste containing 5 wt % ferrite, we were not able to shear off the silver lines because the silver deformed under the shearing tool, due to its softness. Based on the maximum force recorded on the machine, we estimated the shear strength between the modified silver and ferrite to be at least 50 MPa. One reason for the strong bonding was probably the mitigated interfacial stress by the reduced shrinkage mismatch between the modified silver feedstock and ferrite feedstock. The other reason could be that ferrite particles in the modified silver feedstock bonded to the neighboring ferrite at the interface after sintering, which helped improve the interface bonding between silver and ferrite.

Shown in Table 1 are the resistivity values measured on the various silver paste materials after sintering at 930 °C for 2 h. For comparison, the bulk value is also listed. The electrical resistivity from the as-received silver was found to be about 1.2 times of that of bulk silver. After adding 5 wt % and 10 wt % of ferrite powder, the electrical resistivities were 1.8 and 1.9 times of the bulk value, respectively. These values are lower than those of most other metal pastes and inks developed for extrusion-type 3D-printing [35,36,37,38,39].

Table 2 shows the relative permeabilities of the sintered silver samples determined by FEA simulations of the inductor samples with assumed relative permeabilities that match the measured inductances. Both silver materials had relative permeabilities around 1, meaning that the addition of 5 wt % of ferrite powder into silver did not significantly affect the magnetic property of the winding material. This is important since an increase in the winding magnetic permeability would decrease its skin depth [40], leading to an increase in AC winding loss at high frequencies [41].

**Figure 5:**
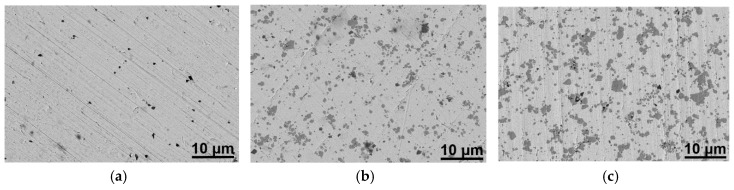
SEM images of 930 °C-sintered (**a**) as-received Ag paste, (**b**) Ag feedstock with 5 wt % ferrite, and (**c**) Ag feedstock with 10 wt % ferrite.

Finally, shown in Figure 5 are scanning electron microscope (SEM) images of sintered silver pastes with the different fractions of ferrite powder. Although the sintering took place at 930 °C, only 30 °C below the melting point of silver, there were still pores present in the sintered, pure silver paste. This may explain its 20% higher electrical resistivity than that of bulk silver. Figure 5b,c shows the microstructures of the sintered silver with ferrite. Comparing 5b and 5c to 5a, it is apparent that the ferrite addition had almost no effect on the sintered density. We also point out that the ferrite phase did not form a percolated network, even with 10 wt % ferrite powder, as shown in Figure 5c. These images may explain the insignificant effect of adding ferrite in the pure silver paste on the electrical resistivity and magnetic permeability shown in Table 1 and Table 2.

## 4. Conclusions

We developed a material feedstock system for a multi-extrusion 3D printer to enable additive manufacturing of ferrite magnetics used in high-frequency power converters. A pure silver paste was tailored by adding small amounts of a NiCuZn ferrite powder to make it printable and co-sinterable with NiCuZn ferrite paste. With the addition of 5 wt % ferrite powder, the densification shrinkage profile of the silver feedstock closely matched that of ferrite, resulting in co-sintered silver and ferrite parts with no obvious warpage, cracks, or interface delamination. The addition of ferrite improved the metal-to-ceramic adhesion with a shear strength of at least 50 MPa. Additionally, adding small fractions (<10 wt %) of ferrite did not significantly increase the electrical resistivity and magnetic permeability of the winding material. Our study shows the feasibility of co-printing and co-sintering ferrite feedstock with a modified silver feedstock to make ferrite inductors. This enables fabrication of novel designs of ferrite magnetic components by 3D-printing to increase the power density as well as efficiency of high-frequency power converters. Our future work will evaluate 3D-printing and co-firing ferrite inductors/transformers with unique core and winding structures.

## Figures and Tables

**Table 1:** Electrical Resistivities of the Various Sintered Ag Pastes and Bulk Ag.

Types of Ag	Average Electrical Resistivity of Three Samples (Ω·m)	Standard Deviation
Bulk Ag (reference)	1.59 × 10^−8^	\
As-received Ag after sintering	1.96 × 10^−8^	0.056
Sintered Ag feedstock with 5 wt % ferrite	2.87 × 10^−8^	0.026
Sintered Ag feedstock with 10 wt % ferrite	3.07 × 10^−8^	0.097

**Table 2:** Relative Permeabilities of the Various Sintered Ag Pastes.

Types of Ag	Average Relative Permeability of Three Samples	Standard Deviation
As-received Ag after sintering	1	0.1
Sintered Ag feedstock with 5 wt % ferrite	0.96	0.15
